# Ecological and morphological correlates of visual acuity in birds

**DOI:** 10.1242/jeb.246063

**Published:** 2024-01-18

**Authors:** Eleanor M. Caves, Esteban Fernández-Juricic, Laura A. Kelley

**Affiliations:** ^1^ University of California Santa Barbara, Department of Ecology, Evolution, and Marine Biology, Santa Barbara, CA 93106, USA; ^2^ University of Exeter, Centre for Ecology and Conservation, Penryn, Cornwall TR10 9FE, UK; ^3^ Purdue University, Department of Biological Sciences, West Lafayette, IN 47907, USA

**Keywords:** Spatial resolution, Light level, Habitat complexity, Diet, Visual ecology

## Abstract

Birds use their visual systems for important tasks, such as foraging and predator detection, that require them to resolve an image. However, visual acuity (the ability to perceive spatial detail) varies by two orders of magnitude across birds. Prior studies indicate that eye size and aspects of a species' ecology may drive variation in acuity, but these studies have been restricted to small numbers of species. We used a literature review to gather data on acuity measured either behaviorally or anatomically for 94 species from 38 families. We then examined how acuity varies in relation to (1) eye size, (2) habitat spatial complexity, (3) habitat light level, (4) diet composition, (5) prey mobility and (6) foraging mode. A phylogenetically controlled model including all of the above factors as predictors indicated that eye size and foraging mode are significant predictors of acuity. Examining each ecological variable in turn revealed that acuity is higher in species whose diet comprises vertebrates or scavenged food and whose foraging modes require resolving prey from farther away. Additionally, species that live in spatially complex, vegetative habitats have lower acuity than expected for their eye sizes. Together, our results suggest that the need to detect important objects from far away – such as predators for species that live in open habitats, and food items for species that forage on vertebrate and scavenged prey – has likely been a key driver of higher acuity in some species, helping us to elucidate how visual capabilities may be adapted to an animal's visual needs.

## Teaser

**Summary:** Visual acuity (the ability to resolve detail) in birds varies with foraging habitat and diet, suggesting that detecting important objects from afar has been a key driver of higher acuity in some species.

## INTRODUCTION

Visual acuity, the ability to resolve static spatial detail, dictates what details can and cannot be resolved in a given scene. Acuity is therefore an important visual parameter in a wide range of visual tasks, such as object detection and recognition, foraging, navigation and communication (Cronin et al., 2014). Acuity is also highly variable across species, varying over at least four orders of magnitude (Caves et al., 2018). Within an eye, areas of higher and lower acuity (such as foveae in camera eyes and acute zones in compound eyes) can be adapted to either the structure of the environment or specific tasks (see Cronin et al., 2014; Hughes, 1977; Land and Nilsson, 2002). Across species, however, few studies have investigated how acuity relates to a species' ecology or environment (but see Veilleux and Kirk, 2014, for mammals; Caves et al., 2017, 2023, for fish; and Land, 1997, for insects). In this study, we examined correlations between acuity and several aspects of ecology in birds (Aves). Birds are an excellent study system for comparative work on acuity for a variety of reasons. First, they are highly visual animals: in the majority of extant bird species, vision is the primary sense (Martin, 2017). Second, birds occupy a diverse range of visual habitats and engage in a variety of visually guided behaviors. Third, acuity varies 30-fold among birds, and the highest known acuities in any species are found in raptorial birds (Caves et al., 2018; Land and Nilsson, 2002). Despite this, we have only a limited understanding of the evolutionary pressures that underlie variation in acuity in birds, or how acuity varies with avian species' morphology and ecology.

Based on the optical properties of eyes, the primary factor that correlates with variation in acuity is eye size. One aspect of an eye's morphology that dictates acuity is the angular width of the region that is viewed by each photoreceptor, which can be thought of as the sampling stations of the eye, and which mediate the first stage in the visual processing pathway. A photoreceptor's angular width is given by the diameter of the photoreceptor divided by the focal length of the eye; thus, longer focal lengths translate to smaller angular resolution, which imparts sharper vision (higher acuity) (McIlwain, 1996). Photoreceptors that collect light over a smaller area, however, suffer from reduced sensitivity (Land and Nilsson, 2002), leading to a fundamental tradeoff between acuity and sensitivity. One way to overcome this tradeoff, i.e. to increase either acuity or sensitivity without reducing the other, is to increase the eye's focal length, which can be accomplished by increasing the size of the eye. In line with this, acuity has been shown to positively correlate with eye size across highly diverse taxa with both camera-type and compound eyes (Caves et al., 2018), as well as within specific groups, including fish (Caves et al., 2017), mammals (Veilleux and Kirk, 2014) and birds (Kiltie, 2000).

Beyond eye size, increasing evidence in vertebrates suggests that visual acuity is driven by the density or receptive field size of retinal ganglion cells (RGCs), rather than photoreceptor density. Although the photoreceptors (rods and cones) are the cells that detect light in vertebrates, and thus as the eye's sampling stations their density carries spatial information, RGCs process visual information further along in the processing pathway than do photoreceptors. Thus, as an anatomical measure, the density of RGCs may provide more appropriate indicators of acuity than does photoreceptor density (Devries and Baylor, 1997; Enroth-Cugell and Robson, 1966; Lee and Stevens, 2007). Therefore, many studies that map RGC densities across an eye use the highest density of RGCs present in the eye to calculate a species' acuity.

Of course, species live in a variety of different environments and have different ecologies, and thus face different perceptual challenges, some of which require higher acuity while others do not. Two particularly relevant perceptual challenges when considering acuity are object detection and resolution. Acuity gives an indication of the distance from which an individual can resolve an object with enough visual information to make a behavioral decision (Kiltie, 2000), so object detection, in particular the need to detect items at large distances, is hypothesized to drive higher acuity (Fernández-Juricic, 2012; Martin, 2017). Support for this comes from insectivorous bats, in which acuity varies with foraging technique; specifically, gleaning species that use vision alone to detect prey have higher acuity than aerial-hawking species that combine vision with ultrasonic echolocation (Eklöf et al., 2014). In birds, some species' foraging methods require visual detection at great distances (for example, a wedge-tailed eagle, *Aquila audax*, searching for rodent prey while soaring high above the ground; Billerman et al., 2022), while others require visual detection at close range (such as a house sparrow, *Passer domesticus*, scratching and pecking in the dirt; Billerman et al., 2022), and still others rely more on other senses than on vision to forage (such as a brown kiwi, *Apteryx australis*, foraging in the leaf litter largely based on its olfactory senses; Billerman et al., 2022). Here, we hypothesized that species that use foraging techniques that require localizing food from further away would have higher acuity than those that search for food from closer distances.

Another perceptual challenge that might relate to visual acuity is object localization. Different species forage for food items that vary in the visual demands required to localize them, from tiny seeds to larger fruits to large vertebrates and carrion, and from immobile (e.g. fruits and flowers) to highly mobile (e.g. invertebrates). Specializing on different food types has been shown to be correlated with acuity, for example in mammals (Veilleux and Kirk, 2014), reef fish (Collin and Pettigrew, 1988a,b), insects (Land, 1997) and elasmobranchs (Litherland and Collin, 2008), in which predatory species, which tend to forage on more mobile prey that is more difficult to localize, on average have higher acuity than non-predatory species. Thus, we hypothesized that species that forage for different types of food would have different acuities.

Several aspects of an organism's habitat can also relate to acuity. Because of the resolution–sensitivity trade-off described above, the light level in which a species operates is hypothesized to vary with its visual acuity. Specifically, for a given eye size, species that live in darker habitats or are active at lower light levels (e.g. are nocturnal) are hypothesized to have increased need for sensitivity, and thus lower acuity, than those that live in lighter habitats (Land, 1990). Habitat spatial complexity is also predicted to have an influence on visual acuity (as it does, for example, in ray-finned fishes; Caves et al., 2017). In birds in particular, one hypothesis is that species in open habitats (such as an ostrich on grassland or a shearwater soaring over the open ocean) should have higher acuity than species in denser, more vegetative habitats, given that important visual stimuli, e.g. aerial predators, are visible from greater distances in open habitats (Fernández-Juricic, 2012). Using eye size as a proxy for acuity, one study of 97 species found indirect support for this hypothesis, in that birds in open habitats had larger eyes than those in complex habitats (Møller and Erritzøe, 2010). However, a larger study of eye size in one-third of terrestrial avian species found the opposite, that species in forested and understory habitats have larger eyes (Ausprey, 2021). Thus, support for this hypothesis is currently mixed. One reason for this may be that larger eyes do not necessarily confer higher acuity, as they can be an adaptation for increased sensitivity. Thus, studies that use direct measures of acuity, rather than eye size as a proxy for acuity, can aid our understanding of how acuity varies with habitat.

Here, we synthesized available literature on visual acuity in birds and then examined relationships between acuity and the factors that we discuss above. First, we calculated the relationship between acuity and eye size, represented by eye axial length, which are known to be correlated (e.g. Kiltie, 2000), but here using the largest sample of birds to date. The purpose of re-examining a well-established relationship was to improve our understanding of how acuity and eye size scale in birds, which may facilitate future comparisons with groups such as mammals (Veilleux and Kirk, 2014) and fish (Caves et al., 2017), and to help researchers who lack behavioral or anatomical data on acuity in their study species, but who have eye size data, to easily extrapolate an acuity value. We then examined how acuity relates to five ecological variables, relating to diet (specifically diet composition, prey mobility and foraging mode), the light level in which a species primarily operates, and habitat.

## MATERIALS AND METHODS

### Comparative database of acuity

We assembled a database of visual acuity in birds using published data; for each species, we recorded the highest reported acuity value, which in the vast majority of studies was the mean of the highest acuities measured across individuals. We then restricted the database to include only data measured using behavioral assays [optomotor assays (see Caves et al., 2020) or conditioned responses] or anatomical methods (specifically studies that estimate acuity from the highest density of RGCs and a measure or assumption of the eye's focal length). We did not include in our database acuity measurements collected using electrophysiological methods or lens optics.

Both anatomical and behavioral methods of measuring acuity have advantages and disadvantages. Unlike anatomical methods, behavioral methods are able to account for diffraction and other optical imperfections, spatial and temporal summation, and higher-order visual processing, and thus some have argued that behavioral measures are a better indicator of an eye's true acuity than those derived anatomically (Arrese et al., 1999). Additionally, behavioral experiments can be carried out at different light levels to account for the fact that acuity varies with the ambient illumination, whereas RGC-based estimates refer only to resolution at high light levels. However, factors such as diffraction and lens aberrations, which can affect acuity, are not likely to vary significantly at least across diurnal bird species, as diurnal species tend to have similar eye shapes and also are not vision limited by their sensitivity to ambient light (Fernández-Juricic, 2012). Behavioral measures can be confounded by factors such as variation in motivation or acclimation to experimental environment. Additionally, in species with more than one area of specialization, as can occur in birds which have two foveas (e.g. Fite and Rosenfield-Wessels, 1975), behavioral assays may require an animal to fixate on a stimulus using an area of the visual field that does not correspond to the area of peak RGC density, resulting in an inaccurate estimate of acuity (see Pettigrew et al., 1988).

To determine whether it would be appropriate to combine acuity data derived from RGCs and behavioral assays for analyses, we performed two analyses. First, we compiled measures of acuity in 28 vertebrate species with camera eyes (the type of eye found in birds) in which acuity has been measured using both methods (Table S2). We found that behaviorally derived and RGC-derived acuity measures from the same species are highly correlated (*P*<0.0001; Fig. S1) in a phylogenetically corrected model using a tree from timetree.org (Kumar et al., 2022). However, this analysis included only five species of birds; thus, to address this issue in a larger dataset of bird acuities, we created a phylogenetic generalized least squares (PGLS) regression in which acuity was the response variable and eye size and method of acuity measurement, and their interaction, were predictors. A phylogenetic ANCOVA (see below for details on the phylogenetic tree used) showed that, because the interaction term between eye size and method of measurement was not significant (*P*=0.39), the slope of the regression line between acuity and eye size was similar for the two methods of measurement. Thus, we concluded that it was appropriate to include both RGC-derived and behaviorally derived measures of acuity in our database for analyses.

For five species, the database included acuity estimates from both behavioral assays and RGC density (with the average difference between the behaviorally and RGC-derived estimates being only 0.84 cycles per degree, cpd – see below). Given the very small differences in acuity obtained from the two methods, and the analyses above regarding combining behavioral and RGC data together, we preferentially used estimates from RGC density in analyses if both behavioral and RGC-based measures of acuity existed. If multiple studies had used the same method to measure acuity in a given species, we used the acuity estimate from the most recent study. Species were only included in the database for analysis if we could locate both eye axial diameter and body mass data for that species (see below), resulting in a sample size of 94 species (Table S1).

Here, we refer to acuity throughout in units of cycles per degree (cpd), which is the number of pairs of black and white stripes an organism can discriminate within a single degree of visual angle. Higher cpd values indicate the ability to resolve finer spatial details, and thus higher acuity. In some of the original literature we surveyed, acuity was reported in alternative units (such as minutes of arc or degrees); prior to inclusion in our database, we translated these values to cpd.

### Phylogenetic relatedness and phylogenetic signal (λ)

To account for phylogenetic relatedness between species, we used a published phylogeny (Burleigh et al., 2015) of 6714 avian taxa which used a 29-locus supermatrix to build a phylogeny with branch lengths. The larger tree was trimmed to include only the 94 species in our acuity database, maintaining branch length information in our sub-tree. The degree of phylogenetic signal in acuity was estimated by calculating Pagel's λ (Freckleton et al., 2002; Pagel, 1999) using the ‘phylosig’ function from the *phytools* package (Revell, 2012). Pagel's λ ranges from 0 (no covariance between trait and phylogenetic structure) and 1 (complete covariance between trait and phylogenetic structure). A likelihood ratio test was used to determine the significance of Pagel's λ against the null hypothesis that λ=0.

### Eye size and body mass

Where possible, we recorded eye axial length (hereafter ‘eye size’) in our database as reported in the original citation; this yielded data on eye size in 73 species. For the remainder, we located published eye size values from a variety of sources (see Table S1), to maximize the number of species for which we had analyzable data. It was uncommon for studies to report the body mass of the individuals used in acuity measurements; therefore, to obtain comparable body mass data for all of the species in our database, we used values from the *CRC Handbook of Avian Body Masses* (Dunning, 2007).

### Classifying species according to ecology

We examined the relationship between visual acuity and several aspects of a species' ecology. Given that many bird species can inhabit a wide array of habitat types, or make use of a diversity of food sources, it can be complex to categorize birds by factors such as diet and habitat; thus, our categories were relatively broad.

#### Habitat

We examined how acuity relates to two aspects of habitat: spatial complexity and light level. To relate acuity to habitat spatial complexity, we used the EltonTraits database (Wilman et al., 2014), which details for all extant bird species the percentage of time spent foraging in eight different habitat types. First, we summed certain percentages to calculate the percentage of time spent in spatially complex habitats (understory and mid-high vegetative habitats); open habitats (aerial and open water habitats); and horizon-dominated habitats (ground, water's surface and at or just above the canopy). The distribution of these percentages revealed that species could roughly be split into two broad classes: those that spent at least 70% of their time in one habitat type (complex, horizon-dominated or open), and those that did not have any clear designation as to a primary habitat type. Therefore, we assigned any species that spends greater than 70% of its time in a given habitat class to that class; species that did not have a score greater than 70% for a given category were labeled as habitat generalists.

To relate habitat light level to acuity, we classified species using habitat descriptions provided in the Cornell Lab of Ornithology Birds of the World database (Billerman et al., 2022). Species were classed as operating in ‘low’, ‘medium’ or ‘high’ light environments based upon their habitat and their activity pattern. Habitats with low light level included forest understories, mangroves, dense shrubland or heathland; species were also included in this category if it was noted that they favor dense habitats. High light level habitats included deserts, grassland, savannah, farmland, steppe, meadow, pelagic oceans, open Antarctic islands, flat beaches or dunes, or mudflats. Medium light level habitats included forest edge, secondary forest or scrub forest; species were also included in this category if they were noted as favoring ‘semi-open’ habitats. Any species that was identified as primarily nocturnal in Wilman et al. (2014), or forages at pelagic depths great enough to be equivalent to nocturnal habitat (Martin, 2017), was classified as inhabiting ‘low’ light habitat; this included all of the owls in our dataset, as well as the king penguin, *Aptenodytes patagonicus*, and Manx shearwater, *Puffinus puffinus*.

#### Diet and foraging

To understand how acuity relates to diet, we first used the EltonTraits database (Wilman et al., 2014), which assigns species to a ‘dominant’ diet category based on the summed scores from 10 constituent diet categories. These categories, which we related to acuity, were plants (including plants, seeds, fruits and nectar), invertebrates and vertebrates (including scavenged prey). Following the designations in the EltonTraits database, any species which had a score of less than 50% for all of the prior categories was classified as an Omnivore. Species were also classified by whether their primary prey type was mobile or immobile prey. Mobile prey included vertebrates and invertebrates (excluding scavenged prey); immobile prey included plant matter of all kinds, such as fruits, seeds, nectar, flowers and fruits, as well as scavenged prey.

Lastly, we classified species by foraging mode using information in the Cornell Lab of Ornithology Birds of the World database (Billerman et al., 2022). Specifically, species were classified as using foraging modes that involve resolving and targeting prey from a distance (far-sighted) versus from close up (near-sighted) foraging maneuvers. Following Ausprey (2021), distance maneuvers included aerial chase, pursuit diving, scavenging and sallying, while close-up foraging maneuvers included gleaning, pecking, dabbling, kicking/scratching and probing.

### Statistical analyses

To statistically analyze how acuity is related to the suite of ecological variables described above, we used a PGLS model run using the gls function from *ape* (Paradis and Schliep, 2019) and a variance–covariance structure based on a Brownian model of evolution. We first used the pairs.panels function of the *psych* package (Revelle, 2019) to examine whether any of our predictors – both continuous and categorical – were highly correlated with one another (corr >0.7) and thus should be dropped, but none were. We then built a series of PGLS models that included acuity as the response variable and every combination of eye size and all of the ecological variables we explored as predictors. We ranked models based on the Akaike information criterion (AIC; Akaike, 1974; Burnham and Anderson, 2002), and then assigned ΔAIC values by calculating the difference between the AIC value of a given model and the AIC value of the best-fit model (i.e. that with the lowest AIC value in that set). Following Burnham et al. (2011), ΔAIC values were used to calculate relative likelihoods for each model *i* within a set using the formula *l_i_*=exp[−(1/2)Δ*_i_*]. We then calculated the probability that each model, *w_i_*, within a set of models, is the best by dividing the likelihood of a given model *l_i_* by the sum of the likelihoods of all models within that set (Burnham et al., 2011).

We then explored the results of the full model by examining each of the categorical ecological variables in turn. In addition to acuity, and following Caves et al. (2017), we explored two additional metrics that give an idea of the importance of vision, and of spatial resolving power, for a given species. First, we calculated ‘relative eye size’ by extracting the residuals from a PGLS model of body mass versus eye size, which can be interpreted as a single measure for each species of whether their eyes are larger or smaller than expected based on their body mass. Species with a positive ‘relative eye size’ have larger eyes than expected for their body mass, whereas species with a negative value have smaller eyes than expected for their body mass. We then ran a PGLS regression on acuity versus relative eye size, and extracted the residuals from that relationship. These residuals, which we term ‘residual acuity’ show whether a given species has even higher or lower acuity than would be expected based on its investment in eye size; in essence, these residuals represent the portion of variation in acuity that is unexplained by variation in relative eye size, and help us to examine acuity without the confounding effects of eye size.

We used *emmeans* (Lenth et al., 2019) to calculate estimated marginal mean acuity, relative eye size and residual acuity in each ecological category. We then used phylogenetic ANOVA (*phytools*; Revell, 2012) with *post hoc* tests corrected for multiple testing (*n*=2, *n*=3 or *n*=4 comparisons depending on the ecological category) using the Holm–Bonferroni correction (Holm, 1979) to examine whether differences between ecological categories were statistically significant when controlling for phylogeny.

All analyses were run using R version 4.0.3 (http://www.R-project.org/). Prior to statistical analyses, acuity, eye size and body mass were log-transformed to improve the normality of the residuals. Using Cook's distance (Cook, 1977), three species (the wedge-tailed eagle, *A. audax,* the Egyptian vulture, *Neophron percnopterus*, and the barn owl, *Tyto alba*) were found to be statistical outliers based on the relationship between acuity and eye size, and so were excluded from further analyses. We ran all of the above analyses first on a complete dataset (including both behavioral and RGC-derived acuity), and second on a dataset comprising only RGC data, though because of low sample sizes we lacked statistical power to run our analyses on a behavior-only dataset.

## RESULTS

### Acuity across birds

**Fig. 1.**
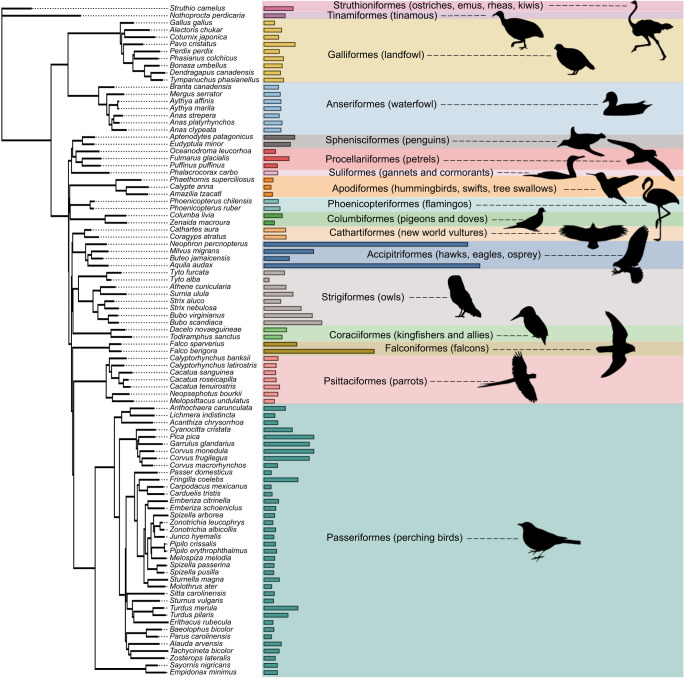
**The phylogenetic distribution of visual acuity in 94 species of birds.** Bars represent acuity in cycles per degree (cpd). Icons (from phylopic.org) show a representative member of each order. Tree pruned from a 6714-taxon tree built by Burleigh et al. (2015).

Using the complete database of published acuity values from 94 species, acuity was highly variable across birds, with the lowest acuity (4.6 cpd in the Anna's hummingbird, *Calypte anna*) being two orders of magnitude lower than the highest acuity (143 cpd in the wedge-tailed eagle, *A. audax*). Mean acuity (±s.d.) was 15.8±20.8 cpd. Pagel's λ (the degree of phylogenetic signal) was estimated to be 0.99, which a likelihood ratio test showed was significantly different from λ=0 (*P*<0.0001). Thus, there was significant phylogenetic signal in acuity, indicating that shared phylogenetic history contributes strongly to trends in acuity across birds (Fig. 1). After excluding statistical outliers, analyses were run on 91 species (*n*=71 species with RGC-derived acuity measures and *n*=20 with behaviorally derived acuity measures) from 38 avian families and 17 avian orders.

### Acuity, eye size and body size

**Fig. 2.**
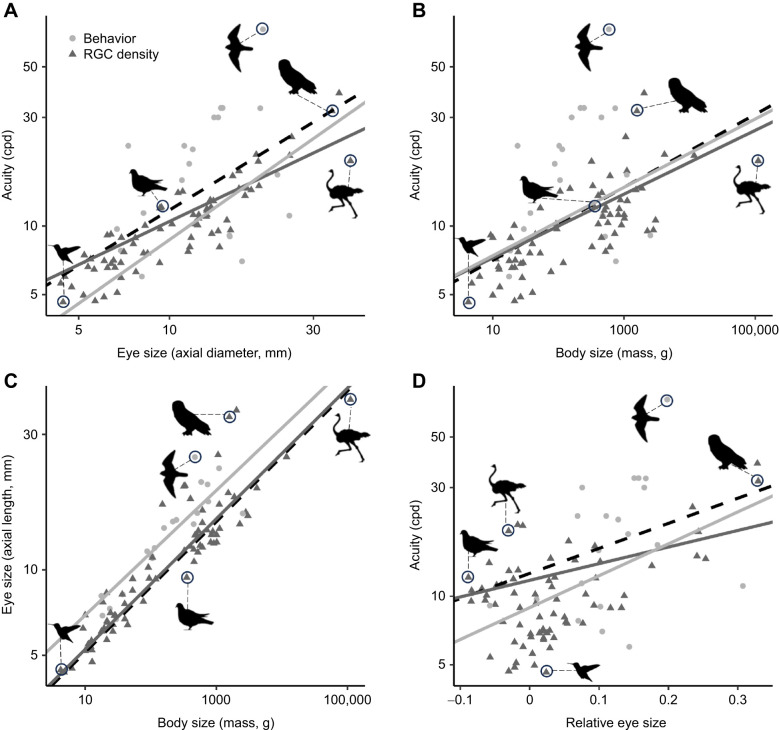
**Phylogenetically corrected relationships between acuity, eye size, body mass and relative eye size in birds.** Data are for a behavior-only dataset (circles), a retinal ganglion cell (RGC)-only dataset (triangles) and a combined dataset (dashed black line) for 91 species of birds. Panels show phylogenetic generalized least squares (PGLS) regressions of (A) acuity versus eye size, (B) acuity versus body mass, (C) eye size versus body mass, and (D) acuity versus relative eye size (which was calculated using the residuals from the regression line in C). Icons show, from left to right in A: Anna's hummingbird, *Calypte anna* (lowest acuity and lowest body mass); rock dove, *Columba livia* (lowest relative eye size); brown falcon, *Falco berigora* (highest acuity); great horned owl, *Bubo virginianus* (highest relative eye size); and common ostrich, *Struthio camelus* (largest eye size and highest body mass).

As expected, PGLS regressions revealed significant, positive relationships between acuity and both eye size (coef.±s.e.=0.81±0.15, *t*_91_=5.51, *R*^2^=0.56, *P*<0.0001; Fig. 2A) and body mass (coef.±s.e.=0.16±0.04, *t*_91_=4.10, *R*^2^=0.46, *P*=0.0001; Fig. 2B). Thus, on average, as eye size or body mass increases, acuity increases. However, extracting the residuals from the PGLS regression of acuity on eye size and examining the relationship between those residuals and body mass yielded no significant relationship (coef.±s.e.=−0.15±0.30, *t*_91_=−0.51, *P*=0.62). Together, these results show that eye size is a strong predictor of acuity, and that the observed correlation between acuity and body mass results from the correlation between eye size and body mass, which themselves are significantly correlated (coef.±s.e.=0.23±0.01, *t*_91_=17.7, *R*^2=^0.93, *P*<0.0001; Fig. 2C). There was also a positive, significant relationship between relative eye size and acuity (coef.±s.e.=1.10±0.34, *t*_91_=3.24, *R*^2^=0.42, *P*=0.002; Fig. 2D), indicating that species with higher relative eye size (larger eyes than expected based on their body size), on average exhibit higher acuity.

When considering acuity derived from RGC density and acuity derived from behavior separately, relationships between acuity and eye size, body size and relative eye size were always in the same direction as for the complete dataset, but were only significant for the RGC-derived dataset (acuity and eye size: coef.±s.e.=0.63±0.09, *t*_71_=7.08, *P*<0.0001; acuity and body mass: coef.±s.e.=0.14±0.02, *t*_71_=5.54, *P*<0.0001; acuity and relative eye size: coef.±s.e.=0.74±0.25, *t*_71_=2.93, *P*=0.005), and not the behavioral dataset (acuity and eye size: coef.±s.e.=0.93±0.60, *t*_20_=1.54, *P*=0.14; acuity and body mass: coef.±s.e.=0.15±0.16, *t*_20_=0.95, *P*=0.35; acuity and relative eye size: coef.±s.e.=1.42±1.15, *t*_20_=1.23, *P*=0.24). However, the lack of significance in the behavioral dataset is likely due to the low sample size (*n*=20 species), as phylogenetic ANCOVA showed no significant differences in the slope of the regression line between acuity and eye size (*P*=0.39), acuity and body size (*P*=0.49), or acuity and relative eye size (*P*=0.06) for the behavioral versus RGC datasets. The relationship between eye and body size was significant for the behavior-only and RGC-only datasets (behavior only: coef.±s.e.=0.22±0.03, *t*_20_=6.71, *P*<0.0001; RGC only: coef.±s.e.=0.23±0.01, *t*_71_=17.07, *P*<0.0001).

### Relationship between acuity and ecology

**Fig. 3.**
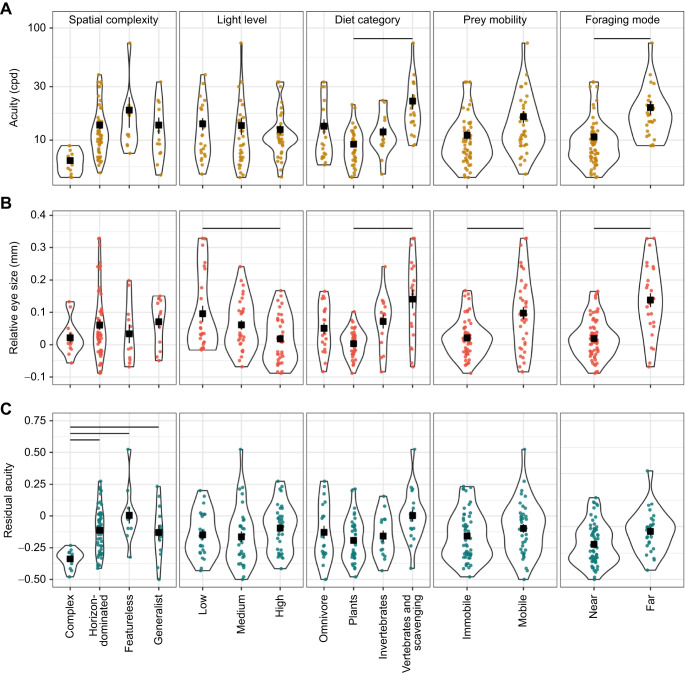
**Acuity, relative eye size and residual acuity across ecological categories in birds.** (A) Acuity, (B) relative eye size and (C) residual acuity data for 91 species of birds. Points show raw data for individual species; black squares show mean and vertical bars show s.e.m. Horizontal black bars connect categorizations that differed significantly (phylogenetic ANOVA; statistical details are given in Results).

To examine the effects of all of our ecological predictors and eye size on acuity together, we first constructed phylogenetically corrected PGLS models that included all combinations of our ecological variables (habitat spatial complexity, light level, diet category, prey mobility and foraging mode) and eye size as predictor variables and acuity as the response variable, and used AIC to rank the models based on fit. Then, to further examine acuity across ecological categories, and why only certain ecological factors significantly predicted acuity in the full model above, we used phylogenetic ANOVA to examine how raw acuity, relative eye size and residual acuity vary across ecological categories while controlling for species relatedness (Fig. 3). We also performed the same analyses using a dataset comprising only acuity data measured using RGC density (Fig. S2), though we lacked statistical power to perform the analyses on a dataset with only behavioral data.

Although the best-fit model (model weight=0.67) included only eye size, a model that also included foraging classification as a predictor had a ΔAIC value of 1.81 relative to the best fit, and a model weight of 0.27, indicating it has substantial support. No other models had a ΔAIC value of less than 6 relative to the best-fit model, or had a model weight higher than 0.03. When using a dataset comprising only acuity measured using RGC density, the results were similar: the best-fit model still included only eye size, though two other models – one additionally including light level, and one additionally including foraging mode – had ΔAIC values of less than 4 relative to the best-fit model, and thus some support. When eye size was not included as a predictor, the best-fit model included only foraging classification (model weight=0.66), and no other parameters appeared in models with a ΔAIC of less than 6 relative to the best-fit model. The same was found when using a dataset comprising only RGC data.

Raw acuity, eye size and body mass across all ecological categories are detailed in Table 1; statistical results for pairwise comparisons within each ecological category are detailed in Table 2. We found that raw acuity (Fig. 3A) was significantly higher in species whose diets consist of vertebrates and scavenged food than in those that eat plant matter (*P*=0.05). Acuity was also higher in species that forage via modes that involve resolving prey from far versus near (*P*=0.04). There were no significant differences in raw acuity across different categories of habitat spatial complexity, habitat light level or prey mobility (Table 2). The same results were found when considering only RGC data, except that, additionally, acuity was significantly higher in species that eat vertebrates than in those that either eat invertebrates (*P*=0.01) or are omnivorous (*P*=0.01) (Fig. S2).

**Table 1.**
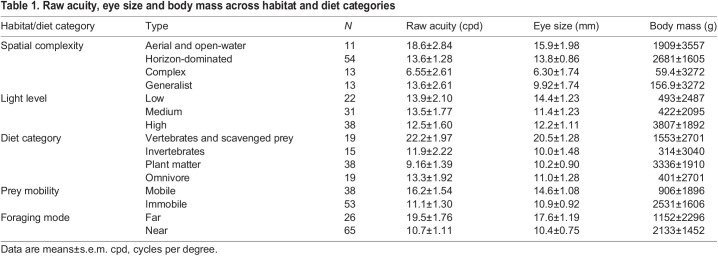
Raw acuity, eye size and body mass across habitat and diet categories

**Table 2.**
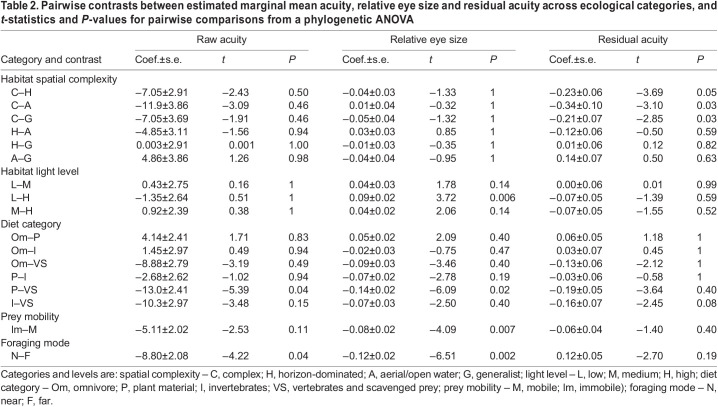
Pairwise contrasts between estimated marginal mean acuity, relative eye size and residual acuity across ecological categories, and *t*-statistics and *P*-values for pairwise comparisons from a phylogenetic ANOVA

However, raw acuity does not account for variation in eye size, so we next examined how relative eye size varies across ecological categories (Fig. 3B), as relative eye size shows how much larger or smaller a species' eyes are than expected based on their body mass. We found that: species in low-light habitats had significantly higher eye investment than those in high-light habitats (*P*=0.009); species that eat vertebrates and scavenged food have higher eye investment than species that eat plants (*P*=0.02); species that eat mobile prey have higher relative eye size than those that eat immobile prey (*P*=0.007); and species that forage from a distance have higher relative eye sizes than those that forage close up (*P*=0.002). There were no significant differences in relative eye size across habitats of different spatial complexity (Table 2). Again, results when examining only RGC-derived acuity were identical, except that additionally we found that species that eat vertebrates or scavenge have significantly higher relative eye size than omnivores (*P*=0.04; Fig. S2).

Examining relative eye size as above can give an indication of how important vision is for a species in a given ecological category, but larger eyes than expected could contribute to increased sensitivity to light, rather than acuity. Therefore, to relate relative eye size directly to acuity, we calculated residual acuity for each species by extracting the residuals from a regression between relative eye size and acuity (Fig. 3C). There were no significant differences in residual acuity across light level, diet category, prey mobility or foraging mode categories (Table 2). We did, however, find that species inhabiting complex environments had lower residual acuity than those in horizon-dominated habitats (*P*=0.04) or aerial and open-water habitats (*P*=0.05). Species inhabiting complex environments also had lower residual acuity than did habitat generalists (*P*=0.04), but not when using an RGC-only dataset (*P*=0.27) (Fig. S2).

## DISCUSSION

**Fig. 4.**
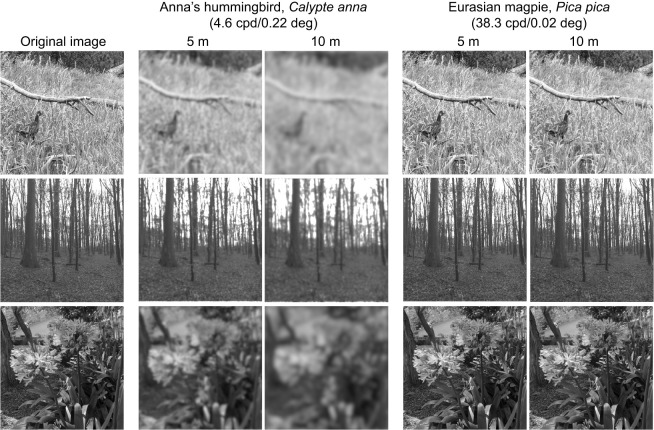
**Portraying avian visual acuity in the perception of natural scenes.** From top to bottom: a bird in a grassy field, a forest and flowers, chosen to represent natural scenes and objects at a variety of spatial scales, from viewing distances of 5 and 10 m. These scenes have been modified using the R package *AcuityView* (Caves and Johnsen, 2017), which uses Fourier methods to delete spatial frequencies from an image that are below the acuity of a given viewer from a given viewing distance. Here, we have portrayed scenes based on the lowest measured acuity in birds (Anna's hummingbird, *Calypte anna*) and the highest non-raptorial acuity (Eurasian magpie, *Pica pica*). Although raptors have the highest known acuity in birds, *AcuityView* can only portray scenes based on acuity lower than that of humans. The visual acuity (cpd) and minimum resolvable angle (deg) are indicated for each species, as well as the assumed viewing distance. Images shown are the green color channel extracted from a full color image, as acuity is achromatic. Photo credit: E.M.C.

This is the largest examination to date of how acuity relates to morphology and ecology in birds. Acuity varies over two orders of magnitude within birds, resulting in interspecific differences in which spatial details can be resolved in a given scene (Fig. 4). As expected based on previous work, we found a strong, positive correlation between acuity and eye size. This finding is in line with predictions based on the optics of camera-type eyes: in camera-type eyes, the angle between photoreceptors, the inter-receptor angle, can be estimated by dividing the distance between photoreceptors by the focal length of the eye (the distance from the point in the lens through which light passes without being bent, to the image on the retina). Thus, larger eyes, which have longer focal lengths, also have smaller inter-receptor angles, translating to higher acuity. The *R*^2^ value for the phylogenetically corrected relationship between acuity and eye size was 0.56, implying that eye size alone explains more than half of the variation in acuity; however, we also found evidence that several aspects of a species' ecology relate to acuity.

### Acuity and habitat

We found that raw acuity did not differ across either habitat spatial complexity or light level categories, though residual acuity was significantly higher in species inhabiting aerial and open-water and horizon-dominated habitats, as well as habitat generalists, than in those in complex habitats. How can we explain the presence of significant differences in only residual, but not raw, acuity between different habitat types? The fact that relative eye size also did not differ significantly across habitats shows that species in all three habitat types have eye sizes that are, on average, in line with allometric predictions based on their body masses. However, our residual acuity results suggest that species inhabiting complex environments have even lower acuity than one would expect based on their eye investment.

The spatial complexity of the physical environment has previously been shown to have an impact on differences in acuity across species. For example, habitat complexity is correlated with acuity in fishes (Caves et al., 2017), including in reef fishes (Collin and Pettigrew, 1988a,b), cichlids (Dobberfuhl et al., 2005) and elasmobranchs (Litherland and Collin, 2008). Interestingly, however, in fishes, increased habitat spatial complexity is associated with higher acuity, which is opposite to what we found here in birds, with species living in the most spatially complex (vegetative) habitats having the lowest acuity. These differences may be explained by the fact that in aquatic environments, sighting distance is constrained by the optics of the medium (water) long before it is constrained by acuity (Johnsen, 2012), whereas in terrestrial environments, acuity can be a primary factor influencing the distance at which objects, such as predators, are detected (Tisdale and Fernández-Juricic, 2009). For species that forage in vegetative habitats, predators may only be visible from relatively short distances. By contrast, species that forage on the ground (which here we term ‘horizon-dominated’) might be predicted to have higher acuity, in order to resolve predators approaching at a distance.

Regarding light level, we found no significant differences in either raw or residual acuity across levels. Although we saw that species in low-light habitats have significantly higher relative eye size than those in high-light habitats, the fact that residual acuity did not differ suggests that any increased investment in eye size likely does not serve to increase the eye's acuity. A recent study of eye size across more than 2700 species of birds found that species that live in dark understory and forest habitats (which here were classified as closed environments) had larger eyes than species that lived in more open habitats (Ausprey, 2021); however, larger eye size does not necessarily translate to higher acuity. Our results, which use direct measures of acuity rather than eye size as a proxy, suggest that particularly in relatively dark environments such as forests, larger eyes may be specialized for increased sensitivity to light, rather than for higher acuity.

Another factor that dictates the light level in which a species operates is activity time, i.e. whether a species is nocturnal, diurnal or crepuscular. Species that are nocturnal are predicted to maximize sensitivity over acuity, and thus to have lower acuity than diurnal birds with similarly sized eyes. In our dataset, the only nocturnal species for which we also had acuity data were seven species of owls, meaning we lacked statistical power to compare acuity in nocturnal versus diurnal birds. An informal comparison, however, showed that average acuity is lower in owls (21.9 cpd) than in diurnal orders with similar eye sizes and foraging styles (i.e. visually from a distance), including hawks and eagles (Accipitriformes; 24.9 cpd) and falcons (Falconiformes; 47.4 cpd), lending support to the hypothesis that activity time may also shape acuity through trade-offs with sensitivity.

### Acuity and diet

We found higher raw acuity in species that eat vertebrates and scavenge, compared with those that eat plant matter. We also found that relative eye size was higher in species that eat vertebrates and scavenge compared with those that eat plants. Species that eat vertebrates or that scavenge for prey thus have higher acuity, and also larger eyes than expected for their body size, but beyond that they do not have higher acuity than predicted based on the size of their relatively large eyes alone. Raw acuity was also higher in species whose foraging maneuvers involve resolving prey from a distance (as species that scavenge and perhaps those that eat vertebrates likely do) than those whose foraging strategies involve resolving prey from close up. Although the most familiar example of this might be the raptorial birds that search for vertebrate prey from far above the ground, and whose acuities are some of the highest ever measured in animals, this pattern also held among smaller species. For example, the black phoebe, *Sayornis nigricans* (8.9 cpd), and least flycatcher, *Empidonax minimus* (8.9 cpd), both of which ‘sally’ or leap from branches to catch flying insects at a distance, have higher acuity than sparrows with similarly sized eyes that scratch and peck the ground to find food (e.g. white-throated sparrow, *Zonotrichia albicollis* 7.7 cpd; white-crowned sparrow, *Zonotrichia leucophrys* 5.9 cpd; house sparrow, *Passer domesticus* 4.9 cpd; and house finch, *Carpodacus mexicanus* 4.7 cpd).

Higher acuity in predatory versus non-predatory species has been shown previously in other groups, including reef fish (Collin and Pettigrew, 1988a,b), mammals (Veilleux and Kirk, 2014), insects (Land, 1997) and elasmobranchs (Litherland and Collin, 2008), although across a large sample of ray-finned fishes, Caves et al. (2017) did not find any association between acuity and diet. In birds, our results together suggest that for species that eat vertebrates or scavenged prey, the need to resolve prey objects may be a powerful force shaping visual function, specifically higher acuity, but that rather than being driven by the mobility of prey items, the more important factor may be the need to detect them from a distance (Tyrrell and Fernández-Juricic, 2017). This is supported by previous work on passerine birds that forage on the ground, which has suggested that species consuming food close to their bills may not require higher acuity (Dolan and Fernández-Juricic, 2010).

### Other factors shaping eyes and acuity

In our dataset, relative eye size differed across numerous ecological categories, including light level, diet, prey mobility and foraging mode. Thus, how large or small eyes are relative to bodies varies with many aspects of ecology, underscoring the fact that eyes are of course shaped by a number of selection pressures beyond visual acuity, including the developmental and energetic costs associated with eyes (Niven and Laughlin, 2008), and their numerous functions beyond resolving an image. For example, species with the highest relative eye sizes were all owls, suggesting that large eyes relative to body size may be most important in nocturnal species that require highly sensitive vision. Some of the lowest relative eye investment was found in the flamingos *Phoenicopterus chilensis* and *Phoenicopterus ruber*, filter feeders that may not need to invest in large eyes given their ability to often forage non-visually (Martin, 2012).

Residual acuity, or how much higher acuity is than expected based on relative eye size, was highest in the brown falcon, *Falco berigora*, but the highest residual acuity excluding falcons was found in the corvids. Although we did not classify corvids as foraging visually from a distance, given that many forage primarily on the ground using a variety of techniques, it is possible that some are indeed relying on distance vision, as they do occasionally soar when foraging (although at lower altitudes than raptors such as eagles), highlighting the complexities of assigning species as flexible as corvids to categories. Additionally, they are highly intelligent birds that use vision in social and cognitive tasks. For example, tool use has been shown to shape aspects of the visual system in New Caledonian crows (Troscianko et al., 2012), implying that the perceptual demands of cognitive tasks may potentially represent other, underexplored, drivers of visual acuity.

Eyes must also, among other things, resolve motion, and although visual acuity is no doubt an important component of motion perception, motion detection may not vary in a clear-cut manner with acuity across species. This idea is supported by our finding that whether or not prey are mobile does not seem to be the key factor underlying the higher acuity seen in species that eat vertebrates, as we found no significant differences in raw or residual acuity between species that eat mobile versus immobile prey. This could arise from the fact that detection of mobile prey may be largely a factor of motion perception, rather than visual acuity. Motion perception and visual acuity arise as a result of different retinal processes. While acuity arises from RGC receptive fields, motion vision is attributed to the photoreceptor type known as double cones (e.g. Goldsmith and Butler, 2005; von Campenhausen and Kirschfeld, 1998), which send information through to RGCs and trigger a pattern of activity across the optic tectum, resulting in the perception of object motion. Additionally, the visual processes by which individuals locate mobile versus immobile prey may be different. Searching for mobile prey items may rely on visual tracking, whereby attention is focused on an object for a longer period of time, as opposed to visual search, where individuals scan their surroundings using fast saccadic eye or head movements to locate immobile food items such as seeds (see Fernández-Juricic, 2012, for a discussion), though some species likely use both visual tracking and visual search techniques, depending on the prey, as is the case in hawks and eagles, which search for both live prey and carcasses.

### Limitations of the study

One limitation of this study is that acuity can be measured in several ways, each of which has advantages and disadvantages (as discussed in the Introduction). Here, however, we attempted to ensure that using acuity data derived in different ways would have minimal impacts on our results by demonstrating that, in species with camera eyes that have had acuity measured using both behavioral and anatomical methods, the two measures are highly correlated.

Another limitation arises from the need to use acuity values as reported in the literature for large, comparative studies such as this one. The majority of studies on acuity report only a single value as representative of a species' acuity, usually the peak acuity measured at any point in the eye. In reality, however, the density of both photoreceptors and ganglion cells, and thus acuity, varies across the retina (e.g. Querubin et al., 2009), with some species possessing two foveas (e.g. Fite and Rosenfield-Wessels, 1975), meaning that different portions of the visual field are viewed with different acuities. Additionally, in some species, the topography of photoreceptors and RGCs can differ between the right and left eyes, as occurs in some parrot species (e.g. Coimbra et al., 2014; Hart et al., 2000; Mitkus et al., 2014), implying that acuity may be lateralized, and differ between eyes. In our literature search, we found only a handful of studies that reported any information about variation in acuity either between eyes or within a single eye. Future studies – especially those which utilize retinal ganglion cell maps, and thus have access to data about variation across the visual field – should report how acuity might vary across the eye.

Lastly, although we include data on more than 90 species in this study, this still represents only a tiny fraction of the total diversity of birds. Thus, studies should continue to measure visual acuity in new species, and analyses such as those conducted here should be revisited, to continue to add to our understanding of the morphological and ecological factors underlying variation in acuity across birds.

### Conclusions

A great deal of work on the function of ganglion cell distribution patterns in birds has identified two primary drivers: foraging, specifically the detection of food items, and predator detection (Martin, 2017). Selection on adaptations to increase the efficiency of foraging may be especially strong in birds, as birds are particularly constrained by the need to have both high power output and low body weight (King and King, 1980). To increase foraging efficiency, higher acuity may be favored especially in species that forage on prey that may be far away. Similarly, early detection of predators – spotting them while they are still at a distance – is key to increasing the likelihood of avoiding a predation attempt (Fernández-Juricic, 2012), so selection on vision to achieve this aim is likely strong. Overall, our findings support these ideas, specifically that a key factor influencing visual acuity in birds is the need to detect objects at a distance: our results support the hypothesis that higher acuity will be selected for in species in open habitats, perhaps for predator detection, and in species that forage on vertebrates and scavenged prey, perhaps to detect their prey from a distance.

## Supplementary Material

10.1242/jexbio.246063_sup1 Supplementary information
