# Supplementary material for: Ecological and morphological correlates of visual acuity in birds
Source: J Exp Biol. 2024 Jan 18;227(2):jeb246063. doi: 10.1242/jeb.246063 (PMC10906485; doi:10.1242/jeb.246063)
Supplement: Supplementary information [file jexbio-227-246063-s1.pdf]

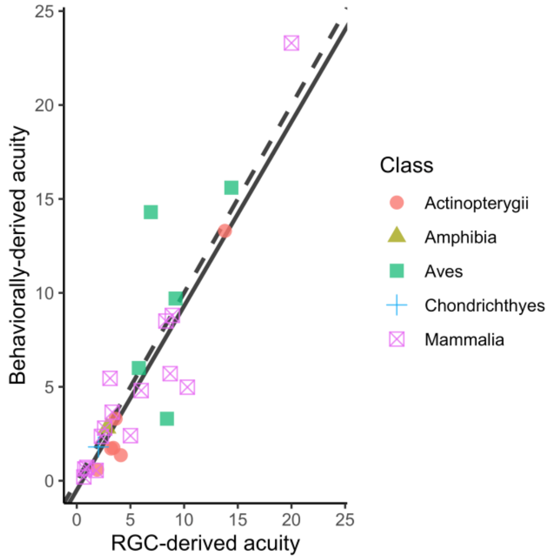

Fig. S1. Correlation between behaviorally- and RGC-derived acuity in 28 vertebrate species. The solid line indicates the predicted relationship if behaviorally- and RGC-derived acuity align perfectly (slope of 1) and the dashed line is the best-fit line from a phylogenetically-corrected (PGLS) regression. Acuity in cycles per degree on both axes.

Journal of Experimental Biology • Supplementary information

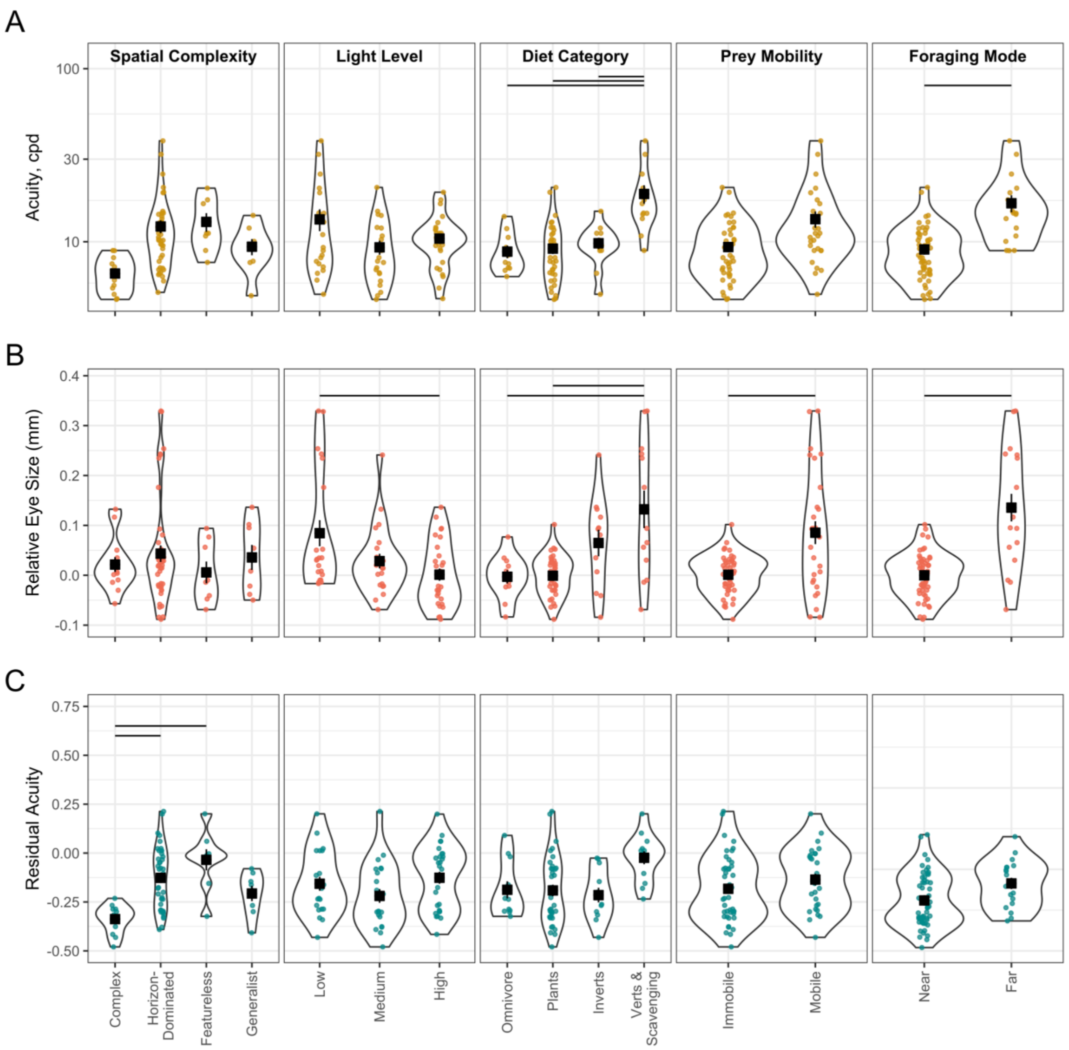

Fig. S2. (A) Acuity, (B) relative eye size, and (C) residual acuity across ecological categories in birds, using only acuity data measured using the density of retinal ganglion cells. Points show raw data for individual species; black squares show mean and vertical black bars show standard error. Horizontal black bars connect categorizations that differed significantly.

Journal of Experimental Biology • Supplementary information

Table S1. Complete database for analysis of acuity across 93 species of birds, listed in the taxonomic order given by the IOC World Bird List, version 13.2 [1]. Columns are: Common name, scientific name (again given by IOC World Bird List), synonyms (listed if they are used either by BirdTree.org, from which the phylogenetic tree used here was generated, or in the original acuity publication), acuity in cycles per degree (cpd), method of acuity measurement, eye axial length (EL, mm), body mass (BM, g), and categorizations for ecological variables [Spatial Complexity (Complex=C, HD=Horizon-Dominated, AO=Aerial/Open Water, G=Generalist); Light Level (L=Low, M=Medium, H=High), Diet Category (Om=Omnivore, P=Plant Material, I=Invertebrates, VS=Vertebrates and Scavenged prey); Prey Mobility (M=Mobile, Im=Immobile); Foraging mode (N=Near, F=Far)]. (*) indicates updated taxonomic name, i.e. the name reported in the original publication is different from that presented here.

Common Name	Scientific Name	Synonym	Acuity (cpd)	Method	ED mm	BM (g)	For-aging Mode	Prey Mobil-ity	Diet Type	Habitat Light Level	Spatial Com-plexity	Citation (acuity, eye size)
**Struthioniformes**												
Common Ostrich	*Struthio camelus*		19.3	RGC	39.8	111000	N	Im	P	H	HD	[2],[2]
**Tinamiformes**												
Chilean Tinamou	*Nothoprocta perdicaria*		14	RGC	10.7	458	N	Im	Om	H	HD	[3],[3]
**Anseriformes**												
Canada Goose	*Branta canadensis*		9.6	RGC	15.9	2812	N	Im	P	H	HD	[4],[4]
Northern Shoveler	*Spatula clypeata*	*Anas clypeata*	11.2	RGC	12	613	N	M	I	H	HD	[5],[5]
Gadwall	*Mareca strepera*	*Anas strepera*	10.1	RGC	12.4	916	N	Im	P	H	HD	[5],[5]
Mallard	*Anas platyrhynchos*		11.9	RGC	13.3	843	N	M	Om	H	HD	[5],[5]

Journal of Experimental Biology • Supplementary information

Greater Scaup	*Aythya marila*		11.2	RGC	13.3	1005	N	M	I	H	AO	[5],[5]
Lesser Scaup	*Aythya affinis*		11.2	RGC	12.5	819	N	Im	P	H	AO	[5],[5]
Red-breasted Merganser	*Mergus serrator*		10.8	RGC	12.5	1015	F	M	VS	M	AO	[5],[5]
**Galliformes**												
Ruffed Grouse	*Bonasa umbellus*		12.1	RGC	13.7	531	N	Im	P	M	HD	[6],[6]
Sharp-tailed Grouse	*Tympanuchus phasianellus*		13	RGC	13.7	882	N	Im	P	L	HD	[6],[6]
Spruce Grouse	*Canachites candensis*	*Dendragapus canadensis*	11	RGC	13.2	474	N	Im	P	L	HD	[6],[6]
Grey Partridge	*Perdix perdix*		10.2	RGC	12.1	405	N	Im	P	H	HD	[6],[6]
Common Pheasant	*Phasianus colchicus*		12.9	RGC	13.9	1120	N	Im	P	H	HD	[6],[6]
Indian Peafowl	*Pavo cristatus*		20.6	RGC	19.4	4155	N	Im	P	M	HD	[7],[6]
Red Junglefowl	*Gallus gallus*		7	B	17.4	7512	N	Im	Om	M	HD	[6],[6]
Japanese Quail	*Coturnix japonica*		9.7	RGC	9.33	95	N	Im	P	H	HD	[6],[6]
Chukar Partridge	*Alectoris chukar*		11.7	RGC	13.3	502	N	Im	P	H	HD	[6],[6]
**Apodiformes**												
Long-tailed Hermit	*Phaethornis superciliosus*		6	RGC	4.5	6	N	Im	P	L	C	[8],[8]
Anna's Humming-bird	*Calypte anna*		4.64	RGC	4.44	4	N	Im	P	M	C	[9],[9]

Rufous-tailed Humming-bird	*Amazilia tzacatl*		5.6	RGC	4.36	5	N	Im	P	M	C	[8],[8]
**Columbiformes**												
Rock Dove	*Columba livia*		12.1	RGC	9.4	354	N	Im	P	H	HD	[7],[10]
Mourning Dove	*Zenaida macroura*		6.89	RGC	9.28	119	N	Im	P	M	HD	[11],[10]
**Phoenicopteriformes**												
American Flamingo	*Phoenicopterus ruber*		10.6	RGC	15.5	3032	N	M	Om	H	HD	[12], [12]
Chilean Flamingo	*Phoenicopterus chilensis*		9.5	RGC	14.5	2277	N	M	I	H	HD	[12], [12]
**Sphenisciformes**												
King Penguin	*Aptenodytes patagonicus*		20.4	RGC	25	11731	F	M	VS	H	AO	[13], [13]
Little Penguin	*Eudyptula minor*		17.46	RGC	17	1108	F	M	VS	H	AO	[13], [13]
**Procellariiformes**												
Leach's Storm Petrel	*Hydrobates leucorhous*	*Oceanodroma leucorhoa*	7.6	RGC	8.2	37	N	M	Om	H	AO	[14], [14]
Northern Fulmar	*Fulmarus glacialis*		16.6	RGC	16.2	612	F	M	VS	H	AO	[14], [14]
Manx Shearwater	*Puffinus puffinus*		8.9	RGC	11.8	453	F	M	VS	H	AO	[14], [15,16]
**Suliformes**												
Great Cormorant	*Phalacrocorax carbo*		9.1	B	15.8	2529	F	M	VS	H	AO	[17], [18]
**Cathartiformes**												

Journal of Experimental Biology • Supplementary information

Black Vulture	*Coragyps atratus*		14.65	RGC	18.1	1882	F	Im	VS	L	HD	[19], [19]
Turkey Vulture	*Cathartes aura*		14.4	RGC	18.7	1518	F	Im	VS	M	HD	[19], [19]
**Accipitriformes**												
Egyptian Vulture	*Neophron percnopterus*		135	B	22.1	2082	F	Im	VS	H	HD	[20], [18]
Wedge-tailed Eagle	*Aquila audax*		143	B	36.0	3449	F	M	VS	M	HD	[21], [16,21]
Black Kite	*Milvus migrans*		32.9	B	20.0	734	F	Im	VS	H	HD	[22], [18]
Red-tailed Hawk	*Buteo jamaicensis*		16.8	B	22.8	1101	F	M	VS	M	AO	[23], [10]
**Strigiformes**												
Western Barn Owl	*Tyto alba*		3.3	RGC	17.5	403	F	M	VS	L	HD	[24,25], [43]
American Barn Owl	*Tyto furcata**		13.6	RGC	17.8	403	F	M	VS	L	HD	[26], [18]
Burrowing Owl	*Athene cunicularia*		14.6	RGC	17.0	151	F	M	VS	L	HD	[26], [26]
Northern Hawk-Owl	*Surnia ulula*		19.2	RGC	19.7	320	F	M	VS	L	HD	[26], [26]
Snowy Owl	*Bubo scandiacus*	*Bubo scandiaca*	38.3	RGC	36.5	2029	F	M	VS	L	HD	[26], [26]
Great Horned Owl	*Bubo virginianus*		32	RGC	34.6	1576	F	M	VS	L	HD	[26],[10]
Tawny Owl	*Strix aluco*		11.1	B	24.9	472	F	M	VS	L	HD	[27], [18]
Great Grey Owl	*Strix nebulosa*		24.6	RGC	25.4	1062	F	M	VS	L	HD	[26], [26]
**Coraciiformes**												

Journal of Experimental Biology • Supplementary information

Laughing Kookaburra	*Dacelo novaeguineae*		15	RGC	19.8	334	F	M	I	M	HD	[28], [18]
Sacred Kingfisher	*Todiramphus sanctus*		12	RGC	9.30	53	F	M	I	M	G	[28], [18]
**Falconiformes**												
American Kestrel	*Falco sparverius*		21.7	B	11.9	115	F	M	I	H	G	[23],[10]
Brown Falcon	*Falco berigora*		73	B	20.4	587	F	M	VS	M	G	[29],[10]
**Psittaciformes**												
Red-tailed Black Cockatoo	*Calyptorhynchus banksii*		9.27	RGC	13.8	722	N	Im	Om	L	G	[30], [30]
Carnaby's Black Cockatoo	*Zanda latirostris*	*Calypto-rhynchus latirostris*	8.09	RGC	14.7	612	N	Im	P	L	C	[30], [30]
Galah	*Eolophusroseicapilla*	*Cacatua roseicapilla*	8.1	RGC	11.7	325	N	Im	P	M	HD	[30], [30]
Long-billed Corella	*Cacatua tenuirostris*		10.32	RGC	12.2	567	N	Im	P	M	HD	[30], [30]
Little Corella	*Cacatua sanguinea*		7.72	RGC	11.5	524	N	Im	P	M	G	[30], [30]
Bourke's Parrot	*Neopsephotus bourkii*		9.2	RGC	6.4	44	N	Im	P	M	G	[31], [18]
Budgerigar	*Melopsittacus undulatus*		6.9	RGC	6.9	29	N	Im	P	H	HD	[31], [18]
**Passeriformes**												
Black Phoebe	*Sayornis nigricans*		8.88	RGC	7.7	19	F	M	I	H	C	[32], [18]
Least Flycatcher	*Empidonax minimus*		8.9	RGC	6.93	10	F	M	I	M	C	[33], [33]

Journal of Experimental Biology • Supplementary information

Brown Honeyeater	*Lichmera indistincta*		7.16	RGC	5.48	11	N	Im	P	L	C	[34], [34]
Red Wattlebird	*Anthochaera carunculata*		14.19	RGC	11.1	106	N	Im	P	M	G	[34], [34]
Yellow-rumped Thornbill	*Acanthiza chrysorrhoa*		9.12	RGC	6.22	9	N	M	I	H	HD	[34], [34]
Blue Jay	*Cyanocitta cristata*		18.75	B	11.6	88	F	Im	Om	L	G	[35], [36]
Eurasian Jay	*Garrulus glandarius*		30	B	14.1	159	N	M	Om	M	HD	[37], [36]
Eurasian magpie	*Pica pica*		33	B	14.8	217	N	Im	VS	M	HD	[37], [36]
Western Jackdaw	*Coloeus monedula*		33	B	15.0	246	N	Im	Om	H	G	[37], [38]
Rook	*Corvus frugilegus*		30	B	14.5	452	N	M	Om	H	HD	[37], [39]
Large-billed Crow	*Corvus macrorhynchos*		9	B	15.9	513	N	Im	Om	M	HD	[40], [36]
Tufted Titmouse	*Baeolophus bicolor*		6.57	RGC	6.60	22	N	M	I	L	C	[41], [41]
Carolina Chickadee	*Poecile carolinensis*	*Parus carolinensis*	4.97	RGC	5.19	10	N	M	I	L	C	[41], [41]
Eurasian Skylark	*Alauda arvensis*		11.4	B	8.20	37	N	Im	P	H	HD	[42], [42]
Tree Swallow	*Tachycineta bicolor*		10.05	RGC	8.30	21	F	M	I	H	G	[33], [33]
Silvereye	*Zosterops lateralis*		7.46	RGC	5.22	13	N	Im	Om	L	C	[34], [34]
White-breasted Nuthatch	*Sitta carolinensis*		6.83	RGC	6.41	27	N	M	Om	L	C	[41], [41]
Common Starling	*Sturnus vulgaris*		6.29	RGC	7.74	77	N	Im	Om	H	HD	[11], [11]

Common Blackbird	*Turdus merula*		22.5	B	11.2	103	N	Im	Om	M	HD	[42], [42]
Fieldfare	*Turdus pilaris*		15.9	B	11.0	106	N	M	I	M	HD	[42], [42]
European robin	*Erithacus rubecula*		6	B	8.10	18	N	M	Om	M	G	[42], [42]
House Sparrow	*Passer domesticus*		4.88	RGC	6.07	27	N	Im	P	M	G	[11], [11]
Eurasian Chaffinch	*Fringilla coelebs*		22.5	B	7.30	24	F	M	I	L	G	[42], [42]
House Finch	*Haemorhous mexicanus*	*Carpodacus mexicanus*	4.69	RGC	5.67	21	N	Im	P	H	C	[11], [11]
American Goldfinch	*Spinus tristis*	*Carduelis tristis*	5.4	RGC	4.74	13	N	Im	P	H	C	[43], [43]
Yellowhammer	*Emberiza citrinella*		9.7	B	6.70	30	N	Im	P	H	HD	[42], [42]
Common Reed Bunting	*Emberiza schoeniclus*		7.8	B	6.90	18	N	Im	P	H	HD	[42], [42]
Chipping Sparrow	*Spizella passerina*		6.62	RGC	5.37	12	N	Im	P	M	HD	[44], [44]
Field Sparrow	*Spizella pusilla*		6.45	RGC	5.63	13	N	Im	P	H	HD	[44], [44]
American Tree Sparrow	*Spizelloides arborea*	*Spizella arborea*	7.03	RGC	6.08	18	N	M	Om	H	HD	[44], [44]
Dark-eyed Junco	*Junco hyemalis*		6.55	RGC	6.23	20	N	Im	P	M	HD	[44], [44]
White-crowned Sparrow	*Zonotrichia leucophrys*		5.93	RGC	6.91	28	N	Im	P	M	HD	[45], [45]
White-throated Sparrow	*Zonotrichia albicollis*		7.7	RGC	7.06	24	N	Im	P	L	HD	[44], [44]

Journal of Experimental Biology • Supplementary information

Song Sparrow	*Melospiza melodia*		7.07	RGC	6.53	22	N	Im	Om	M	HD	[44], [44]
California Towhee	*Melozone crissalis*	*Pipilo crissalis*	7.59	RGC	8.46	53	N	Im	P	M	G	[45], [45]
Eastern Towhee	*Pipilo erythrophthalmus*		8.35	RGC	7.59	40	N	Im	Om	L	HD	[44], [44]
Eastern Meadowlark	*Sturnella magna*		10.2	RGC	10.2	92	N	M	I	H	HD	[46], [46]
Brown-headed Cowbird	*Molothrus ater*		5.1	RGC	6.71	40	N	Im	P	M	HD	[11], [11]

Journal of Experimental Biology • Supplementary information

Table S2. 28 vertebrate species with camera eyes in which acuity has been measured both behaviorally and using peak retinal ganglion cell density. A (*) indicates a species name that was different in the original publication and has been updated to reflect current taxonomic designations.

Class	Scientific Name	Common Name	Acuity from Behavior (cpd)	Acuity from RGC density (cpd)	Behavior Citation	RGC citation
**Actinopterygii**	*Danio rerio*	Zebrafish	0.59	1.89	[47]	[48]
	Pomacentrus amboinensis	Ambon damelfish	1.36	4.1	[49]	[49]
	Pseudochromis fuscus	Yellow dottyback	1.71	3.2	[49]	[49]
	Rhinecanthus aculeatus	Triggerfish	1.75	3.41	[50]	[50]
	Toxotes chatareus	Largescale archerfish	3.3	3.62	[51]	[51]
	Toxotes jaculatrix	Archerfish	13.3	13.8	[52]	[52]
**Amphibia**	*Lithobates pipiens**	Northern leopard frog	2.8	2.82	[53]	[53]
**Aves**	*Tyto alba*	Barn owl	3.3	8.4	[25]	[54]
	Calypte anna	Anna's Hummingbird	6	5.78	[9]	[9]
	Neopsephotus bourkii	Bourke's parrot	9.7	9.2	[55]	[31]
	Melopsittacus undulatus	Budgerigar	14.3	6.9	[55]	[55]
	Columba livia	Pigeon	15.6	14.4	[56]	[56]
**Chondrichthyes**	*Chiloscyllium punctatum*	Brown-banded bamboo shark	1.8	2.02	[57]	[58]
**Mammalia**	*Myotis daubentonii*	Daubenton's bat	0.2	0.66	[59]	[60]
	Mesocricetus auratus	Golden hamster	0.55	1.8	[61]	[62]
	Tarsipes rostratus	honey possum	0.63	0.75	[63]	[64]
	Carollia perspicillata	Seba's short-tailed bat	0.71	0.94	[65]	[66]
	Tursiops truncatus	Bottlenose dolphin	3.65	3.3	[67]	[68]
	Sminthopsis crassicaudata	Fat-Tailed Dunnart	2.36	2.3	[69]	[69]
	Tupaia belangeri	Northern tree shrew	2.4	5	[70]	[70]

Journal of Experimental Biology • Supplementary information

*Dasyurus maculatus**	Northern native cat	2.8	2.6	[71]	[71]
*Notamacropus eugenii**	Tamar wallaby	4.8	6	[72]	[72]
Bos taurus	Cow	4.98	10.3	[73]	[74]
Orcinus orca	Killer whale (water)	5.45	3.1	[75]	[76]
Phoca vitulina	Harbor seal	5.7	8.7	[77]	[77]
Canis familiaris	Domestic dog	6.2	8.3	[78]	[79]
Aotus azarai	Azara's owl monkey	8.5	8.3	[80]	[81]
Felis catus	Domestic cat	8.8	8.9	[82]	[79]
Equus caballus	Horse	23.3	20	[83]	[83]

Literature Cited

1. Gill, F. *et al.* (2021) *IOC world bird list*, IOC
2. Boire, D. *et al.* (2001) Quantitative analysis of the retinal ganglion cell layer in the ostrich, *Struthio camelus*. *Brain, Behavior, and Evolution* 58, 343–355
3. Krabichler, Q. *et al.* (2015) The visual system of a Palaeognathous bird: Visual field, retinal topography and retino-central connections in the Chilean Tinamou (*Nothoprocta perdicaria*). *Journal of Comparative Neurology* 523, 226–250
4. Fernández-Juricic, E. *et al.* (2011) Testing the terrain hypothesis: Canada geese see their world laterally and obliquely. *Brain, Behavior and Evolution* 77, 147–158
5. Lisney, T.J. *et al.* (2013) Ecomorphology of eye shape and retinal topography in waterfowl (Aves: Anseriformes: Anatidae) with different foraging modes. *Journal of Comparative Physiology A: Neuroethology, Sensory, Neural, and Behavioral Physiology* 199, 385–402
6. Lisney, T.J. *et al.* (2012) Interspecifc variation in eye shape and retinal topography in seven species of galliform bird (Aves: Galliformes: Phasianidae). *Journal of Comparative Physiology A: Neuroethology, Sensory, Neural, and Behavioral Physiology* 198, 717–731
7. Hart, N.S. (2002) Vision in the peafowl (Aves: *Pavo cristatus*). *The Journal of experimental biology* 205, 3925–3935
8. Lisney, T.J. *et al.* (2015) Eye Morphology and Retinal Topography in Hummingbirds (Trochilidae: Aves). *Brain, Behavior and Evolution* 86, 176–190
9. Goller, B. *et al.* (2019) Spatial and temporal resolution of the visual system of the Anna's hummingbird (*Calypte anna*) relative to other birds. *Physiological and biochemical zoology* 92, 481–495
10. Hall, M.I. and Ross, C.F. (2007) Eye shape and activity pattern in birds. *Journal of Zoology* 271, 437–444
11. Dolan, T. and Fernández-Juricic, E. (2010) Retinal ganglion cell topography of five species of ground-foraging birds. *Brain, Behavior and Evolution* 75, 111–121
12. Lisney, T.J. *et al.* (2020) Retinal topography in two species of flamingo (Phoenicopteriformes: Phoenicopteridae). *Journal of Comparative Neurology* 528, 2848–2863
13. Coimbra, J.P. *et al.* (2012) Retinal ganglion cell topography and spatial resolving power in penguins. *Brain, Behavior and Evolution* 80, 254–268
14. Mitkus, M. *et al.* (2016) Vision on the high seas: spatial resolution and optical sensitivity in two procellariiform seabirds with different foraging strategies. *Journal of Experimental Biology* 219, 3329–3338
15. Martin, G.R. (2017) *The sensory ecology of birds*, Oxford University Press
16. Brooke, M.D.L. *et al.* (1999) The scaling of eye size with body mass in birds. *Proceedings of the Royal Society B: Biological Sciences* 266, 405
17. Strod, T. *et al.* (2004) Cormorants keep their power: Visual resolution in a pursuit-diving bird under amphibious and turbid conditions. *Current Biology* 14, 376–377

Journal of Experimental Biology • Supplementary information

18. Ritland, S.M. (1982) *The allometry of the vertebrate eye*, The University of Chicago
19. Lisney, T.J. *et al.* (2013) Comparison of eye morphology and retinal topography in two species of new world vultures (Aves: Cathartidae). *Anatomical Record* 296, 1954–1970
20. Fischer, A.B. (1968) Laboruntersuchungen und freilandbeobachtungen zum sehvermögen und verhalten von altweltgeiern. PhD Thesis
21. Reymond, L. (1985) Spatial visual acuity of the eagle *Aquila audax*: A behavioural, optical and anatomical investigation. *Vision Research* 25, 1477–1491
22. Potier, S. *et al.* (2016) Visual abilities in two raptors with different ecology. *The Journal of Experimental Biology* 219, 2639–2649
23. Mcisaac, H.P. (2001) Raptor acuity and wind turbine blade conspicuity. In *National Avian - Wind Power Planning Meeting IV*, pp. 59–87
24. Harmening, W.M. *et al.* (2009) Spatial contrast sensitivity and grating acuity of barn owls. *Journal of Vision* 9, 1–12
25. Orlowski, J. *et al.* (2012) Night vision in barn owls: visual acuity and contrast sensitivity under dark adaptation. *Journal of vision* 12, 4–4
26. Lisney, T.J. *et al.* (2012) Eye shape and retinal topography in owls (Aves: Strigiformes). *Brain, Behavior and Evolution* 79, 218–236
27. Martin, G.R. and Gordon, I.E. (1974) Increment-threshold spectral sensitivity in the tawny owl (*Strix aluco*). *Vision Research* 14, 615–621
28. Moroney, M.K. and Pettigrew, J.D. (1987) Some observations on the visual optics of kingfishers (Aves, Coraciformes, Alcedinidae). *Journal of Comparative Physiology A* 160, 137–149
29. Reymond, L. (1987) Spatial visual acuity of the falcon, *Falco berigora*: A behavioural, optical and anatomical investigation. *Vision Research* 27, 1859–1874
30. Coimbra, J.P. *et al.* (2014) Topographic specializations in the retinal ganglion cell layer correlate with lateralized visual behavior, ecology, and evolution in cockatoos. *Journal of Comparative Neurology* 522, 3363–3385
31. Mitkus, M. *et al.* (2014) Retinal ganglion cell topography and spatial resolution of two parrot species: Budgerigar (*Melopsittacus undulatus*) and Bourke's parrot (*Neopsephotus bourkii*). *Journal of Comparative Physiology A: Neuroethology, Sensory, Neural, and Behavioral Physiology* 200, 371–384
32. Gall, M.D. and Fernández-Juricic, E. (2010) Visual fields, eye movements, and scanning behavior of a sit-and-wait predator, the black phoebe (Sayornis nigricans). *Journal of Comparative Physiology A* 196, 15–22
33. Tyrrell, L.P. and Fernández-Juricic, E. (2017) The hawk-eyed songbird: retinal morphology, eye shape, and visual fields of an aerial insectivore. *The American Naturalist* 189, 709–717
34. Coimbra, J.P. *et al.* (2014) Topographic specializations in the retinal ganglion cell layer of Australian passerines. *Journal of Comparative Neurology* 522, 3609–3628

Journal of Experimental Biology • Supplementary information

35. Fite, K.V. and Rosenfield-Wessels, S. (1975) A comparative study of deep avian foveas. *Brain, Behavior and Evolution* 12, 97–115
36. Ausprey, I.J. (2021) Adaptations to light contribute to the ecological niches and evolution of the terrestrial avifauna. *Proceedings of the Royal Society B: Biological Sciences* 288, 20210853
37. Dabrowska, B. (1975) Investigations on visual acuity of some corvine species. *Folia Biologica* 23, 311–332
38. Howland, H.C. *et al.* (2004) The allometry and scaling of the size of vertebrate eyes. *Vision Research* 44, 2043–2065
39. Browne, R.G. *et al.* (2007) The schematic eye of the rook, Corvus frugilegus. *Folia Zoologica* 56, 399–404
40. Yamamoto, K. *et al.* (2001) Near-field visual acuity in Japanese jungle crows (*Corvus macrorhynchos*). *Physiology and Behavior* 72, 283–286
41. Moore, B.A. *et al.* (2013) Interspecific differences in the visual system and scanning behavior of three forest passerines that form heterospecific flocks. *Journal of Comparative Physiology A: Neuroethology, Sensory, Neural, and Behavioral Physiology* 199, 263–277
42. Donner, K. (1951) The visual acuity of some passerine birds. *Acta zoologica Fennica* 66
43. Baumhardt, P.E. *et al.* (2014) Do American goldfinches see their world like passive prey foragers? A study on visual fields, retinal topography, and sensitivity of photoreceptors. *Brain, Behavior and Evolution* 83, 181–198
44. Moore, B.A. *et al.* (2015) Vision in avian emberizid foragers: maximizing both binocular vision and fronto-lateral visual acuity. *Journal of Experimental Biology* 218, 1347–1358
45. Fernández-Juricic, E. *et al.* (2011) Visual systems and vigilance behaviour of two ground-foraging avian prey species: White-crowned sparrows and California towhees. *Animal Behaviour* 81, 705–713
46. Tyrrell, L.P. *et al.* (2013) Looking above the prairie: localized and upward acute vision in a native grassland bird. *Scientific Reports* 3, 3231
47. Tappeiner, C. *et al.* (2012) Visual acuity and contrast sensitivity of adult zebrafish. *Frontiers in zoology* 9, 10
48. Pita, D. *et al.* (2015) Vision in two cyprinid fish: Implications for collective behavior. *PeerJ* DOI: 10.7717/peerj.1113
49. Parker, A.N. *et al.* (2017) Comparison of functional and anatomical estimations of visual acuity in two species of coral reef fish. *The Journal of Experimental Biology* 220, 2387–2396
50. Champ, C. *et al.* (2014) Visual acuity in a species of coral reef fish: *Rhinecanthus aculeatus*. *Brain, Behavior and Evolution* 83, 31–42
51. Temple, S.E. *et al.* (2013) A comparison of behavioural (Landolt C) and anatomical estimates of visual acuity in archerfish (*Toxotes chatareus*). *Vision Research* 83, 1–8
52. Ben-Simon, A. *et al.* (2012) Visual acuity in the archerfish: behavior, anatomy, and neurophysiology. *Journal of vision* 12, 18–18
53. Aho, A.-C. (1997) The visual acuity of the frog (*Rana pipiens*). *Journal of Comparative Physiology A* 180, 19–24
54. Wathey, J.C. and Pettigrew, J.D. (1989) Quantitative analysis of the retinal ganglion cell layer and optic nerve of the barn owl *Tyto alba*. *Brain, Behavior, and Evolution* 33, 279–292

55. Lind, O. *et al.* (2012) Luminance-dependence of spatial vision in budgerigars (Melopsittacus undulatus) and Bourke's parrots (Neopsephotus bourkii). *Journal of Comparative Physiology A* 198, 69–77
56. Hodos, W. *et al.* (1991) Age-Dependent Retinal Changes in Visual Acuity Morphology in Pigeons. *Vision research* 31, 669–677
57. Ryan, L.A. *et al.* (2016) Visual resolution and contrast sensitivity in two benthic sharks. *The Journal of Experimental Biology* 219, 3971–3980
58. Lisney, T.J. and Collin, S.P. (2008) Retinal ganglion cell distribution and spatial resolving power in elasmobranchs. *Brain, Behavior and Evolution* 72, 59–77
59. Eklöf, J. (2003) Vision in echolocating bats. Göteborg University, Göteborg, Sweden
60. Cechetto, C. *et al.* (2020) Retinal ganglion cell topography and spatial resolving power in echolocating and non-echolocating bats. *Brain, Behavior and Evolution* 95, 58–68
61. Emerson, V.F. (1980) Grating acuity of the golden hamster. *Experimental Brain Research* 38, 43–52
62. Y-C, T. and Blakemore, C. (1976) Regional specialization in the golden hamster's retina. *Journal of Comparative Neurology* 168, 439–457
63. Arrese, C. *et al.* (2002) Visual capabilities in a crepuscular marsupial, the honey possum (*Tarsipes rostratus*): a visual approach to ecology. *Journal of Zoology* 256, 151–158
64. Dunlop, S.A. *et al.* (1994) The retinal ganglion cell layer and optic nerve in a marsupial, the honey possum (Tarsipes rostratus). *Brain, behavior and evolution* 44, 307–323
65. Suthers, R. (1966) Optomotor responses by echolocating bats. *Science* 152, 1102–1104
66. Heffner, R.S. *et al.* (2007) Sound-localization acuity and its relation to vision in large and small fruit-eating bats: I. Echolocating species, Phyllostomus hastatus and Carollia perspicillata. *Hearing Research* 234, 1–9
67. Herman, L.M. *et al.* (1975) Bottle-nosed dolphin: double-slit pupil yields equivalent aerial and underwater diurnal acuity. *Science* 189, 650–652
68. Mass, A.M. and Supin, A.Y. (1995) Ganglion cell topography of the retina in the bottlenosed dolphin, Tursiops truncatus. *Brain, Behavior and Evolution* 45, 257–265
69. Arrese, C. *et al.* (1999) Retinal structure and visual acuity in a polyprotodont marsupial, the fat-tailed dunnart (*Sminthopsis crassicaudata*). *Brain, behavior and evolution* 53, 111–26
70. Petry, H.M. *et al.* (1984) Spatial contrast sensitivity of the tree shrew. *Vision research* 24, 1037–1042
71. Harman, A.M. *et al.* (1986) Visual acuity of the northern native cat (*Dasyurus hallucatus*) - Behavioural and anatomical estimates. *Behavioural Brain Research* 22, 211–216
72. Hemmi, J.M. and Mark, R.F. (1998) Visual acuity, contrast sensitivity and retinal magnification in a marsupial, the tammar wallaby (*Macropus eugenii*). *Journal of Comparative Physiology - A Sensory, Neural, and Behavioral Physiology* 183, 379–387
73. Entsu, S. *et al.* (1992) Visual acuity of cattle determined by the method of discrimination learning. *Applied Animal Behaviour Science* 34, 1–10

Journal of Experimental Biology • Supplementary information

74. Hebel, R. (1976) Distribution of retinal ganglion cells in five mammalian species (pig, sheep, ox, horse, dog). *Anatomy and Embryology* 150, 45–51
75. White, D. *et al.* (1971) Visual acuity of the killer whale (Orcinus orca). *Experimental Neurology* 32, 230–236
76. Mass, A.M. *et al.* (2013) Ocular anatomy, ganglion cell distribution and retinal resolution of a killer whale (Orcinus orca). *Brain, Behavior and Evolution* 81, 1–11
77. Hanke, F.D. *et al.* (2009) Retinal ganglion cell topography in juvenile harbor seals (Phoca vitulina). *Brain, Behavior and Evolution* 74, 102–109
78. Neuhaus, W. and Regenfuss, E. (1967) The visual acuity of the dog under different brightness. *Zeitschrift für vergleichende Physiologie* 57, 137–146
79. Heffner, R.S. and Heffner, H.E. (1992) Visual factors in sound localization in mammals. *Journal of Comparative Neurology* 317, 219–232
80. Ordy, J. and Samorajski, T. (1968) Visual acuity and ERG-CFF in relation to the morphologic organization of the retina among diurnal and nocturnal primates. *Vision research* 8, 1205–1225
81. Yamada, E.S. *et al.* (2001) M and P retinal ganglion cells of the owl monkey: morphology, size and photoreceptor convergence. *Vision Research* 41, 119–131
82. Hall, S. and Mitchell, D. (1991) Grating acuity of cats measured with detection and discrimination tasks. *Behavioural brain research* 44, 1–9
83. Timney, B. and Keil, K. (1992) Visual acuity in the horse. *Vision Research* 32, 2289–2293
